# A Cross-Sectional Study to Understand COVID-19 Vaccine Hesitancy Among Nursing Students and Health Workers: Implications for Future Outbreak Preparedness in South Africa

**DOI:** 10.3390/ijerph23081042

**Published:** 2026-08-11

**Authors:** Lindokuhle Mokoena, Teboho Abram Moloi, Tanusha Singh

**Affiliations:** 1 Department of Environmental Health, Faculty of Health Sciences, University of Johannesburg, Johannesburg 2006, South Africa; 2 Statistical and Data Science, University of Johannesburg, Johannesburg 2006, South Africa

**Keywords:** vaccine hesitancy, vaccine uptake, vaccination acceptance, disease prevention, risk reduction

## Abstract

**Highlights:**

Although COVID-19 vaccine hesitancy among healthcare workers (HCWs) has been widely reported, evidence on the relationship between knowledge, attitudes, perceptions, and vaccine uptake remains inconsistent.

**Public health relevance—How does this work relate to a public health issue?**

- COVID-19 vaccine hesitancy among HCWs and nursing students may reduce vaccination coverage and potentially compromise workforce preparedness during future infectious disease outbreaks.
- Understanding factors associated with voluntary vaccine uptake is critical for strengthening preparedness for future infectious disease emergencies in South Africa.

**Public health significance—Why is this work of significance to public health?**

- Despite generally positive knowledge and attitudes toward COVID-19 vaccination, concerns about vaccine safety, long-term effects, and mandatory vaccination policies persisted among respondents.
- Age and occupational role were significant predictors of voluntary vaccination uptake, highlighting the importance of demographic-specific approaches to vaccine promotion.

**Public health implications—What are the key implications or messages for practitioners, policy makers and/or researchers in public health?**

- Tailored communication and educational interventions should target younger HCWs and nursing students to address persistent misconceptions, incorrect beliefs arising from misinformation (false or inaccurate information shared irrespective of intent) or personal interpretation, and vaccine-related concerns.
- Future outbreak preparedness strategies should prioritize trust-building, transparent risk communication, and voluntary vaccine engagement to improve vaccine acceptance among healthcare personnel.

**Abstract:**

Background: Vaccine hesitancy is a global challenge affecting workplaces and academic institutions. Despite efforts, low vaccination rates persist in South Africa, increasing the risks associated with pathogen exposure. This study assessed the knowledge, attitudes, and perceptions (KAPs) of healthcare workers (HCWs) and students at a Nursing College regarding voluntary and mandatory COVID-19 vaccination. Methods: A quantitative cross-sectional study was conducted using a structured, self-administered electronic questionnaire. All eligible nursing students and staff at the participating Nursing College were invited to participate. Of the 504 eligible individuals, 372 completed the questionnaire, corresponding to a response rate of 73.8%. Data were analyzed using IBM SPSS Statistics version 30.0. Logistic regression and Mann–Whitney U tests were used to assess associations and differences between groups, with statistical significance set at *p* < 0.05. Results: Most respondents were female (302, 81.0%) and single (252, 67.9%). The age group 26 to 40 was most prominent (144, 38.8%). Only 42.2% of respondents (*n* = 157) were fully immunized with no boosters. Student nurses dominated (344, 92.5%), with 226 (60.9%) having no COVID-19 infections and 198 (72.0%) voluntarily receiving the vaccine. Occupation (*p* = 0.030) and age group (*p* = 0.027) were independently associated with reporting voluntary COVID-19 vaccination. Age was also associated with respondents’ attitudes and perceptions of the COVID-19 vaccine (*p* < 0.001) as well as their understanding of the vaccine (*p* = 0.002). Conclusions: Occupation and age group were independently associated with the reported basis on which COVID-19 vaccination was received (voluntary versus mandatory). Targeted communication and educational strategies addressing these factors may improve vaccine acceptance and preparedness for future outbreaks.

## 1. Introduction

Vaccination has been a cornerstone of global public health for controlling the spread of infectious diseases and preventing outbreaks. Vaccines played a pivotal role in reducing the COVID-19-related morbidity and mortality worldwide and remain an essential component of preparedness for future infectious disease emergencies. Since their introduction, substantial evidence has confirmed their effectiveness in preventing severe disease, hospitalization, and death. However, the emergence of SARS-CoV-2 variants, waning immunity, breakthrough infections, and evolving booster recommendations have shifted attention towards sustaining vaccine confidence and maintaining protection among high-risk populations, including HCWs [1,2,3,4,5,6,7]. However, ensuring high vaccination coverage remains challenging due to varied public opinions on mandatory versus voluntary vaccination policies [8]. Although mandatory vaccination policies may improve vaccine coverage during public health emergencies, they may also generate resistance when perceived as conflicting with individual autonomy, cultural values, or personal beliefs [9]. The principle of informed consent, therefore, remains fundamental to vaccination programs by ensuring that individuals receive accurate information regarding the potential benefits and risks of vaccination before making voluntary healthcare decisions. Examining the impact of such policies within diverse populations with socioeconomic and cultural complexities, like South Africa, provides critical insights into how vaccination strategies might better address both public health goals and individual concerns.

South Africa’s vaccination system operates under the Expanded Program on Immunization (EPI), which historically focuses on childhood vaccines [1,2]. However, adult vaccinations, including influenza, COVID-19 [3], tetanus, human papillomavirus (HPV), and pneumococcal infections [10], are also available, particularly for high-risk populations, through public and private healthcare sectors [11,12]. The South African Department of Health further recommends occupational vaccinations, such as hepatitis A and B, for workers at increased risk of exposure to infectious material, including healthcare, waste management, and food handling [13,14,15]. These measures align with the country’s commitment to reducing the burden of vaccine-preventable diseases and highlight the important role of vaccinations in protecting both workers and the wider community.

South African regulations further support vaccination through several legislative and regulatory frameworks. These include the Occupational Health and Safety Act (OHSA), which requires employers to protect employees from biological hazards by ensuring access to appropriate preventive measures, including vaccination where indicated [16]. The South African Health Products Regulatory Authority (SAHPRA) [17] oversees vaccine availability, safety, and efficacy under the Medicines and Related Substances Act of 1965 [18], while the National Department of Health (NDoH) develops national vaccination guidelines [19]. The National Health Act of 2003 [20] provides the legislative framework for healthcare delivery, including public vaccination programs, while the Mine Health and Safety Act includes provisions for protecting workers in high-risk industries like mining through appropriate occupational health measures [21]. Together, these legislative and regulatory frameworks provide a robust foundation for supporting both voluntary and mandatory vaccination programs, particularly during public health emergencies.

Healthcare workers play a central role in successful vaccination programs because they are both at increased occupational risk of infectious disease exposure and trusted sources of vaccine information for patients and communities. Consequently, their knowledge, attitudes, and perceptions regarding vaccination influence not only their own vaccination behavior but also public confidence in immunization programs [22]. International studies have consistently reported generally favorable attitudes towards COVID-19 vaccination among HCWs, with age, sex, professional experience, employment duration, and vaccine knowledge influencing vaccine acceptance and uptake [23,24,25,26,27,28]. Extensive clinical and post-marketing surveillance has shown that authorized COVID-19 vaccines generally have favorable benefit-risk profiles when used in populations for whom they are recommended [29,30,31,32,33,34,35,36]. Nevertheless, safety risks differ according to vaccine platform, dose, age, sex, and underlying clinical risk. Rare cases of myocarditis and pericarditis have been reported predominantly among adolescent and young adult males following mRNA vaccination, while adenoviral-vector vaccines have been associated with the very rare syndrome of vaccine-induced immune thrombotic thrombocytopenia (VITT), also referred to as thrombosis with thrombocytopenia syndrome (TTS) [37,38,39,40]. Emerging laboratory studies have raised questions regarding residual manufacturing-template DNA, persistence of vaccine-derived material, and changes in antibody subclass profiles following repeated mRNA vaccination; however, findings remain methodologically debated, and their clinical significance has not been established [41]. These recognized adverse events emphasize the importance of informed consent, product-specific recommendations, and continued pharmacovigilance rather than implying that vaccination is entirely without risk [29,37,38,39,40]. Evidence available during the study period also consistently demonstrated that COVID-19 vaccination during pregnancy was safe and effective, reducing the risk of severe maternal disease while contributing to neonatal protection through transplacental antibody transfer, leading to recommendations supporting vaccination during pregnancy [32,33,34,35,36].

As SARS-CoV-2 evolved, booster vaccination became an important component of maintaining protection among HCWs and other high-risk populations. Protection against symptomatic SARS-CoV-2 infection declines progressively over time following primary vaccination, whereas protection against severe disease remains substantially more durable. Consequently, breakthrough infections among vaccinated individuals became increasingly common with waning immunity and the emergence of immune-evasive viral variants [6,42,43,44]. Current international recommendations, therefore, advocate periodic booster vaccination for HCWs according to national public health guidance, circulating variants, and updated vaccine formulations rather than fixed vaccination intervals [4,5]. Although post-marketing surveillance has generally supported the safety of recommended booster doses, continued pharmacovigilance remains essential to detect rare adverse events, evaluate long-term effectiveness, and optimize vaccination strategies for different population groups [4,29,37,38,39,40]. Immunological studies have described changes in spike-specific IgG subclasses following repeated mRNA vaccination; however, the clinical significance of these observations remains uncertain and continues to be investigated [45,46].

Despite extensive evidence demonstrating the effectiveness of COVID-19 vaccines and generally favorable benefit-risk profiles in recommended populations [1,2,3,4,5,6,7], vaccine hesitancy persists among a proportion of HCWs. Initial hesitancy among some HCWs is not uncommon, with 93.3% of HCWs ultimately receiving vaccination and 24.2% initially hesitant but later accepting vaccination after receiving reliable information [47]. Concerns regarding vaccine safety, recognized rare adverse events, long-term effects, misinformation, institutional trust, and mandatory vaccination policies continue to influence vaccination decisions despite generally high vaccine acceptance rates reported internationally [48,49,50,51]. Importantly, studies have demonstrated that many HCWs who were initially hesitant subsequently accepted vaccination after receiving reliable, evidence-based information, highlighting the importance of transparent communication, ongoing professional education, and trust in public health institutions [52]. Trust can be undermined not only by misinformation circulating through social or informal media but also when official recommendations evolve without clear explanation or when emerging evidence necessitates revisions to previous guidance. During a rapidly evolving emergency, changes in guidance may be scientifically appropriate as new evidence becomes available; however, presenting preliminary conclusions with excessive certainty or failing to explain why recommendations have changed may create confusion and weaken confidence in public health institutions [53]. Authorities should therefore communicate what is known, what remains uncertain, the evidence informing recommendations and the reasons for subsequent revisions while openly correcting inaccurate or superseded information [51]. Research from Greece indicates that training HCWs and health science students on evidence-based practices is vital for improving vaccine uptake [54].

Although numerous international studies have examined HCWs’ KAP towards COVID-19 vaccination, considerably less evidence is available from South Africa, where socioeconomic inequalities, cultural diversity, varying healthcare access and differing pandemic experiences may uniquely influence vaccination decisions. Furthermore, relatively few studies have simultaneously examined KAPs regarding both voluntary and mandatory COVID-19 vaccination among HCWs and nursing students within the South African context. For the purposes of this study, ‘knowledge’ refers to the factual understanding of COVID-19 vaccines, ‘attitudes’ refer to the participants’ feelings or evaluations towards vaccination (e.g., support, opposition), and ‘perceptions’ refer to their subjective interpretations or beliefs about the vaccine’s safety, effectiveness, and policies such as mandates. Although attitudes and perceptions are closely related, perceptions represent broader cognitive constructs influenced by personal, social, and contextual factors, whereas attitudes reflect an evaluative position. In a setting characterized by substantial variation in healthcare access, educational attainment and information sources, understanding the factors that shape HCWs’ vaccination decisions is essential for informing future vaccination policies, strengthening health system resilience and improving preparedness for future infectious disease emergencies. Therefore, this study aims to assess the KAPs of South African HCWs and nursing students regarding COVID-19 vaccination, examining both voluntary and mandatory perspectives. The primary objective is to evaluate awareness and factors influencing HCWs’ decisions to vaccinate, while secondary objectives are to analyze vaccination uptake, identify demographic influences, and explore HCWs’ perspectives on vaccine hesitancy.

## 2. Materials and Methods

### 2.1. Study Design

This cross-sectional, quantitative study was conducted to assess the knowledge, attitudes, and perceptions of HCWs regarding voluntary and mandatory COVID-19 vaccination.

### 2.2. Setting

The study was carried out at a Nursing College in Mpumalanga, South Africa. Data were collected over one month, from 1 October to 31 October 2023. All eligible nursing students and staff at the college were invited to participate through an email containing a link to the electronic questionnaire. A reminder email was sent three days after the initial invitation. The questionnaire required approximately 10–15 min to complete. No names, email addresses, or other direct personal identifiers were collected with the questionnaire responses.

### 2.3. Participants and Recruitment

The study population (*n* = 504) comprised healthcare workers (HCWs) and nursing students enrolled in the Diploma in General Nursing (R171) and Degree in General Nursing (R425) programs at the college. Nursing students were included because they undertook supervised clinical placements involving direct patient care and therefore experienced occupational exposure risks relevant to the study. Nevertheless, their professional status differed from that of qualified HCWs and is acknowledged as a limitation. Eligible participants were individuals aged 18 years or older and were enrolled or employed at the college during the study period. All eligible individuals were invited to participate. Participation was voluntary, and respondents who provided informed consent and completed the questionnaire were included in the analysis. Data were collected using a structured, self-administered electronic questionnaire containing closed-ended and Likert-scale items (Supplementary Materials File S1). Of the 504 eligible individuals invited, 372 completed the questionnaire.

### 2.4. Variables

The primary outcome variable was the reported basis on which COVID-19 vaccination was received, categorized as voluntary or mandatory vaccination among respondents who reported receiving at least one dose of a COVID-19 vaccine. Independent variables included demographic characteristics (age, sex, marital status, and occupation), vaccination status, previous COVID-19 infection, KAP regarding COVID-19 vaccination, and reasons for vaccine hesitancy. Potential confounding variables included age, occupation, and previous COVID-19 infection, which were controlled for in the multivariable logistic regression analysis. Only respondents who reported receiving at least one COVID-19 vaccine dose and who indicated whether vaccination was voluntary or mandatory were included in the logistic regression analysis (*n* = 275). Nine vaccinated respondents who did not answer this question were excluded from the regression model.

### 2.5. Data Sources and Measurement

Data was collected using a structured, self-administered questionnaire developed from a literature review and refined with expert input in epidemiology, healthcare, and survey design. The tool comprised four sections: (A) socio-demographic information, (B) ten knowledge items on COVID-19 vaccination, (C) ten attitude and perception items, and (D) six items on reasons for vaccine hesitancy. Responses were rated on a five-point Likert scale (1 = strongly disagree, 5 = strongly agree), with a mean score of 3.0 considered neutral. Scores above 3.0 indicated agreement and below 3.0 disagreement; for reporting, categories were grouped into agreement or disagreement, though the full scale was retained for analysis. Administered in English, the questionnaire demonstrated acceptable to high reliability, with Cronbach’s Alpha values of 0.716 (knowledge), 0.861 (attitudes and perceptions), and 0.754 (hesitancy), confirming consistent measurement across constructs.

### 2.6. Bias

To reduce measurement error, questionnaire items were informed by instruments used in previous studies and were reviewed by experts in epidemiology, healthcare, and survey design. No direct personal identifiers were collected, which may have reduced social desirability concerns. Nevertheless, voluntary participation may have introduced self-selection and non-response bias because individuals with stronger views or greater interest in COVID-19 vaccination may have been more likely to participate. Ethical clearance was granted by the University of Johannesburg Ethics Review Committee (REC 2298-2023), ensuring adherence to ethical research standards.

### 2.7. Study Size

An a priori sample-size calculation was conducted using G*Power version 3.1.9.7 [55]. The calculation assumed an effect size of 0.20, a two-sided significance level of 0.05, and a statistical power of 80%. The estimated minimum required sample was 372 participants. All 504 eligible nursing students and staff were invited to participate to maximize recruitment, and 372 completed the questionnaire, meeting the calculated minimum sample requirement.

### 2.8. Quantitative Variables

All Likert-scale responses were treated as ordinal variables but summarized using means and standard deviations to facilitate interpretation. To enhance clarity in reporting, responses were grouped into three categories: disagreement (mean score < 3.0), neutral (score = 3.0), and agreement (mean score > 3.0). This approach simplified interpretation while retaining the richness of the original five-point scale for statistical analysis.

### 2.9. Statistical Methods

Data was entered, cleaned, and analyzed using IBM SPSS Statistics version 30.0 (IBM Corp., Armonk, NY, USA). Descriptive statistics, including frequencies and percentages, were used to summarize categorical variables, while means and standard deviations were calculated for continuous variables. Normality of the composite KAP scores was assessed using the Kolmogorov–Smirnov test before selecting the appropriate inferential statistical tests. Multivariable logistic regression analysis was performed to identify factors associated with reporting voluntary rather than mandatory COVID-19 vaccination. Because the KAP scores were not normally distributed, non-parametric Mann–Whitney U tests were used to compare these scores across binary demographic groups. Missing data were minimal (<1%) and excluded from relevant analyses without affecting the overall results. All eligible individuals were invited to participate; however, voluntary participation may have introduced non-response and self-selection bias. A *p*-value of <0.05 was considered statistically significant for all inferential analyses.

## 3. Results

### 3.1. Participants

The number and percentage of respondents’ biographical details are shown in Table 1. Of the 504 eligible nursing students and staff invited to participate, 372 completed the questionnaire, corresponding to a response rate of 73.8%. All 372 completed questionnaires were included in the analysis.

### 3.2. Descriptive Data

The majority were nursing students (344, 92.5%), female (302, 81.2%), and single (252, 67.9%) (Table 1). The most common age group was 26–40 years with *n* = 144 (38.8%). A sizeable portion of the respondents (157, 42.2%) were fully vaccinated with no boosters, while 28.1% had received their boosters. A smaller group (6.2%) were awaiting their second vaccination dose and slightly less than a quarter (23.2%) had not been vaccinated. The decision to get vaccinated was mainly left to the individuals (72.0%). Almost two-thirds of the respondents (226, 60.9%) had not had the COVID-19 infection. COVID-19 infection was reported both before and after vaccination, although this cross-sectional study was not designed to evaluate vaccine or booster effectiveness. Less than half of the respondents (154, 41.8%) used electronic media such as television, radio, etc., for COVID-19 and precautionary measures-related information. Nine vaccinated respondents did not indicate whether their vaccination was voluntary or mandatory and were therefore excluded from the logistic regression analysis.

### 3.3. Outcome Data

Table 2 and Table 3 show the frequency count, percentage responses, means, and standard deviations for each of the three constructs, namely knowledge of COVID-19 vaccination (Table 2), attitude and perceptions towards COVID-19 vaccination, and the reasons for hesitating to take COVID-19 vaccination (Table 3). The responses were measured using a 5-point Likert scale, with 1 denoting strong disagreement and 5 denoting strong agreement; however, for ease of interpretation, the two lower and upper scales were combined [25].

Analysis reported in Table 2 demonstrates that eight of the ten statements had more positive responses (mean > 3) towards knowledge of COVID-19 vaccination. In their view, less than half of the respondents (159, 43.2%) strongly agreed/agreed that COVID-19 vaccines are effective at keeping one from getting COVID-19. Many of the respondents (321, 88.7%) strongly agreed/agreed that they maintained the regulations for preventing COVID-19 after vaccination (e.g., social distancing). Several of the respondents (208, 57.5%) strongly agreed that the COVID-19 vaccine will also help keep one from getting seriously ill even if they get COVID-19. Most respondents (290, 80.1%) strongly agreed/agreed that there was a website where one could apply for vaccination in South Africa, and 317 (87.6%) strongly agreed/agreed that, like all other vaccines, the COVID-19 vaccine has the potential for some side effects. Slightly fewer (224, 61.9%) strongly agreed/agreed that vaccines can be given to a person with pre-existing health conditions, and 239 (66.0%) strongly agreed/agreed that if there are vaccine side effects, they normally go away in a few days. Less than half (151, 41.7%) of the respondents strongly agreed/agreed that the COVID-19 vaccine can create long-term physical and health problems.

However, 182 (50.3%) of the respondents strongly disagreed/disagreed that fully vaccinated people are allowed to do everything. Some (143, 39.4%) of the respondents also strongly disagreed/disagreed that the vaccine can be given to pregnant women. Therefore, the summated mean score of 3.56 and standard deviation of 1.78 indicate an overall agreement with the items measuring the knowledge of the COVID-19 vaccination construct.

It is evident from Table 3 that seven items had a more positive response towards the attitudes and perceptions of COVID-19 vaccination, as seen by the mean score that is above 3. Of the respondents that strongly agreed/agreed with the statements, the majority (310, 86.6%) were concerned about the COVID-19 pandemic, 194 (54.2%) thought that the COVID-19 vaccine was safe and effective, 212 (59.2%) thought that if they were eligible for the vaccine, they needed to take it urgently, 302 (81.2%) were aware that their family and neighbors’ should take the vaccine, 270 (72.6%) understood that they should motivate their families and neighbors to take the vaccine, 256 (68.8%) thought the vaccination would stop the spread of the COVID-19 virus, and 172 (46.2%) strongly agreed/agreed that the vaccine should be mandatory for HCWs. The level of agreement with items measuring attitude and perception toward the COVID-19 vaccination construct had an overall mean score of 3.47 and a standard deviation of 1.18.

Slightly less than half of the respondents (182, 48.9%) strongly disagreed/disagreed that they were not well informed about vaccines, 158 (42.5%) strongly disagreed/disagreed that they were hesitant to take the COVID-19 vaccine, and 165 (44.4%) of them also strongly disagreed/disagreed that the vaccine should be mandatory for all South Africans. The reasons for COVID-19 vaccine hesitancy reflect mean scores below 3. Specifically, most of the respondents strongly disagreed/disagreed with various hesitancy factors: 213 (57.3%) regarding ineligibility for the vaccine, 218 (58.6%) about lacking knowledge, and 160 (43.0%) concerning fears of side effects. Additionally, 305 (82.0%) rejected concerns about vaccine costs, 279 (75.0%) disagreed with religious objections, and 184 (50.3%) refuted hesitancy due to other unspecified reasons.

### 3.4. Main Results

The factor structure of constructs related to knowledge of COVID-19 vaccination, attitudes and perceptions towards COVID-19 vaccination, and reasons for vaccine hesitancy were evaluated. The items were subjected to exploratory factor analysis using principal axis factoring. Exploratory factor analysis was performed separately for each construct. Each construct demonstrated a one-factor solution (eigenvalue > 1), indicating that the items measured a single underlying construct [56]. All retained items exhibited factor loadings greater than 0.40, exceeding the recommended threshold for practical significance and supporting their retention in the respective scales [57]. The Kaiser–Meyer–Olkin (KMO) measurement of sampling adequacy was 0.691 for the knowledge construct, 0.842 for attitude and perceptions, and 0.755 for vaccine hesitancy, indicating that the dataset was suitable for factor analysis. Bartlett’s Test of Sphericity further validated this, showing statistical significance (*p* < 0.001), suggesting a moderate to strong association among the items. Reliability was assessed using Cronbach’s alpha coefficients. The knowledge (α = 0.716), attitudes and perceptions (α = 0.861), and vaccine hesitancy (α = 0.754) constructs all demonstrated acceptable to good internal consistency, supporting the reliability of the questionnaire for the present study [58,59].

### 3.5. Logistic Regression Analysis of Factors Influencing COVID-19 Vaccination Decisions

Compared with nursing students, academic and administrative staff had significantly higher odds of reporting voluntary COVID-19 vaccination (aOR = 2.289, 95% CI: 1.083–4.838; *p* = 0.030) (Table 4). Compared with respondents aged 18–40 years, participants aged ≥41 years had significantly lower odds of reporting voluntary COVID-19 vaccination (aOR = 0.629, 95% CI: 0.416–0.949; *p* = 0.027). Furthermore, the results indicate that sex, marital status, vaccination status, infection status, knowledge, attitude and perceptions, and reasons for hesitating to take the COVID-19 vaccine are not associated with voluntarily receiving the COVID-19 vaccine (*p*-value > 0.05).

### 3.6. Demographic Differences in COVID-19 Vaccine Knowledge, Attitudes, and Perceptions

**Figure 1:**
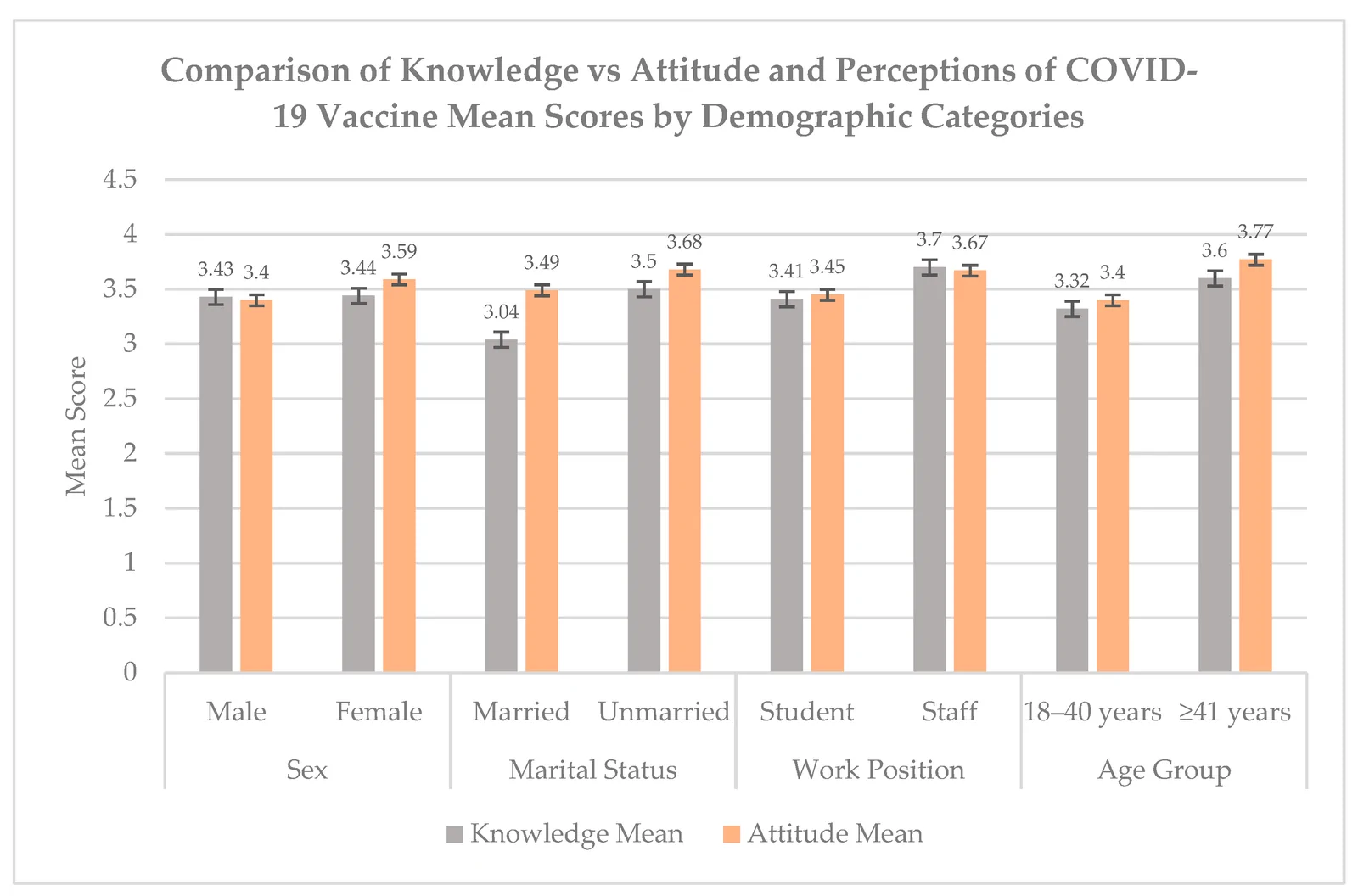
Comparison of mean KAP scores across demographic groups. Statistical comparisons were performed using the Mann–Whitney U test.

To assess demographic differences in knowledge, attitudes, and perceptions of the COVID-19 vaccine, all demographic variables were first recorded into binary categories to facilitate the interpretation of the results. A normality test was performed to examine the distribution of each variable. The Kolmogorov–Smirnov test yielded statistically significant *p*-values, suggesting that the data were not normally distributed and that non-parametric tests were appropriate. Consequently, the Mann–Whitney U test was used to compare demographic groups. The analysis found no significant differences in COVID-19 vaccine knowledge, attitudes, or perceptions across sex, marital status, or job position groups (*p* > 0.05, Figure 1). However, statistically significant differences were observed between the age groups in both attitudes and perceptions (*p* < 0.001) and knowledge towards COVID-19 vaccination (*p* = 0.002). Specifically, respondents aged 41 and older demonstrated a better understanding of the vaccine, with a mean score of 3.77 (SD = 0.87), compared to younger respondents (ages 18–40), who had a mean score of 3.40 (SD = 0.89).

## 4. Discussion

The study sample predominantly consisted of young, single, and largely nursing students, with a notable sex imbalance, comprising 81.2% female and 18.8% male. This demographic profile aligns with global healthcare trends, such as those noted by Karlsson et al. [60], who emphasized the importance of understanding vaccination confidence across different population profiles in the healthcare sector. The sex disparity also mirrors findings from Dror et al. (2020), who reported greater vaccine hesitancy among women [61]. However, more recent evidence suggests that sex differences in vaccine acceptance are influenced by multiple interacting factors, including perceived vaccine safety, risk perception, occupational exposure and trust in public health authorities, rather than sex alone. These findings indicate that demographic characteristics should be interpreted within their broader social and occupational contexts. The predominance of female respondents reflects the demographic composition of the nursing profession in South Africa, where women comprise the majority of practicing nurses and nursing students. The high proportion of single respondents (67.9%) and nursing students (92.5%) further highlights the need to consider demographic factors, such as age, sex, and marital status, when examining vaccine attitudes. These characteristics provide important context for interpreting the study findings because younger HCWs and nursing students are likely to represent the future healthcare workforce and play an important role in promoting vaccine confidence within their communities. Educational attainment and access to reliable health information have consistently been associated with greater vaccine confidence and willingness to vaccinate, reinforcing the importance of strengthening vaccine literacy during professional training [60,61].

**Vaccination Uptake and Attitudes Toward Mandates**

The study revealed a high rate of COVID-19 vaccination uptake among HCWs at the Nursing College, with most participants choosing voluntary vaccination. This finding demonstrates that high vaccine uptake can be achieved without compulsory vaccination and reflects both respect for individual autonomy and a generally high level of vaccine confidence among HCWs. Although vaccine acceptance has varied internationally, more recent evidence indicates that HCWs have generally maintained positive attitudes towards COVID-19 vaccination despite evolving recommendations regarding booster doses, waning immunity, and emerging SARS-CoV-2 variants. This trend reflects respect for individual autonomy while underscoring the high level of vaccine acceptance among HCWs, a finding consistent with Islam et al. (2021), who highlighted similar acceptance rates in community surveys [62]. Furthermore, religious beliefs did not majorly impact vaccine acceptance, with 75% disagreeing that religion posed a barrier. These findings support the study hypothesis that COVID-19 vaccination was generally well accepted among HCWs, particularly when vaccination remained voluntary. While the findings demonstrate strong support for voluntary COVID-19 vaccination among HCWs, they should not be interpreted as endorsing compulsory vaccination policies. Occupational vaccination policies require careful assessment of the specific pathogen, the likelihood and consequences of workplace transmission, evidence that vaccination reduces transmission or severe disease, the availability of less restrictive preventive measures, and the vaccine’s age-, sex-, and product-specific risk profile. Any proposed vaccination requirement should therefore satisfy the ethical principles of necessity, proportionality, equity and least-restrictive intervention; provide appropriate medical exemptions and procedural safeguards; and be accompanied by transparent consultation and access to balanced information regarding benefits, uncertainties and recognized adverse events [8,9,16,20]. The high voluntary uptake observed in this study suggests that strengthening vaccine literacy, institutional trust, convenient access to vaccination and respectful engagement with HCWs may achieve high vaccination coverage without relying primarily on coercive measures.

**Knowledge and Perceptions of COVID-19 Vaccination**

The analysis demonstrated that respondents had a generally positive understanding of COVID-19 vaccination, with 88.7% recognizing the importance of maintaining preventive measures even after vaccination, 80.1% being aware of online vaccine registration, and 87.6% acknowledging potential side effects. However, certain misconceptions persisted: 50.3% disagreed that fully vaccinated individuals had unrestricted movement, and 39.4% were uncertain or disagreed about vaccine suitability for pregnant women. The mean knowledge score (3.56) indicates a generally good level of vaccine knowledge; however, these persistent misconceptions highlight important gaps in understanding specific aspects of COVID-19 vaccination. Misunderstandings regarding post-vaccination preventive measures likely reflect the evolving public health guidance during the pandemic, as recommendations changed in response to emerging variants, breakthrough infections, and increasing evidence of waning immunity. These findings reinforce the importance of providing HCWs with timely, evidence-based updates as scientific knowledge evolves.

Particularly noteworthy was the uncertainty surrounding COVID-19 vaccination during pregnancy. Although substantial evidence accumulated during and after the study period has consistently demonstrated that COVID-19 vaccination during pregnancy is both safe and effective, reducing severe maternal disease while providing passive protection to newborns through transplacental antibody transfer, a sizeable proportion of respondents remained uncertain. This finding represents a gap in participants’ knowledge rather than uncertainty in the scientific evidence available during and after the study period.

**Influence of Demographics on Vaccine Knowledge and Attitudes**

The multivariable logistic regression analysis identified occupation and age group as factors associated with reporting voluntary rather than mandatory COVID-19 vaccination. The Mann–Whitney U analysis further demonstrated significant age-related differences in respondents’ knowledge and attitudes towards COVID-19 vaccination, with older participants demonstrating higher knowledge scores than younger respondents. Older respondents (41 years and above) demonstrated higher knowledge scores (M = 3.60) and more favorable attitudes (M = 3.77) compared to younger participants. This finding may reflect greater professional experience and previous exposure to vaccination programs and infectious disease outbreaks. This finding is consistent with recent evidence demonstrating that demographic characteristics, including age, professional experience and occupational role, remain important determinants of vaccine confidence and acceptance among HCWs [63,64,65]. Older or more experienced HCWs may perceive themselves to be at greater occupational and personal risk of severe COVID-19, while also possessing greater experience with previous vaccination programs and infectious disease outbreaks, contributing to higher levels of vaccine confidence. Conversely, younger HCWs and students may be more susceptible to rapidly evolving information environments, including misinformation disseminated through digital platforms, reinforcing the importance of integrating evidence-based vaccinology and risk communication into undergraduate and professional healthcare education. These findings emphasize that demographic differences should not simply be viewed as predictors of vaccine uptake but as indicators of where targeted educational interventions may be most beneficial. Tailoring vaccine communication strategies according to age, professional experience, and occupational role may therefore strengthen vaccine confidence among HCWs and improve preparedness for future infectious disease emergencies.

**Perceptions of Vaccine Safety and Effectiveness**

Most respondents perceived the COVID-19 vaccine to be safe and effective, consistent with the high vaccination uptake observed in this study. Although relatively few vaccinated respondents reported COVID-19 infection, these findings should be interpreted cautiously because vaccine effectiveness is influenced by factors such as waning immunity, emerging SARS-CoV-2 variants and the timing of vaccine administration. Nevertheless, COVID-19 vaccines continue to provide substantial protection against severe disease, hospitalization, and death, even when breakthrough infections occur. Most respondents agreed that vaccine-related side effects generally resolve within a few days. Such concerns are understandable given the rapid development and deployment of COVID-19 vaccines during the pandemic. However, extensive post-marketing surveillance conducted worldwide has consistently demonstrated favorable safety profiles for COVID-19 vaccines. Recognized adverse events, including rare cases of myocarditis and pericarditis, particularly following mRNA vaccination, and transient menstrual disturbances, have been well characterized, with regulatory authorities consistently concluding that the benefits of vaccination substantially outweigh the risks in recommended populations. Persistent concerns regarding long-term safety therefore appear to reflect ongoing communication challenges rather than deficiencies in scientific evidence. The uncertainty surrounding vaccination among individuals with pre-existing medical conditions and pregnant women further reinforces the need for targeted educational interventions. Current evidence consistently supports COVID-19 vaccination during pregnancy because it reduces severe maternal disease while providing passive protection to infants through transplacental antibody transfer. Continued uncertainty among some HCWs therefore highlights the importance of regularly updating healthcare professionals as evidence evolves. Addressing these concerns through transparent, evidence-based communication is essential for maintaining vaccine confidence, particularly as vaccination strategies continue to evolve in response to emerging variants, updated booster recommendations, and future infectious disease threats.

**Sources of Vaccine Information and Communication Strategies**

The study found that many respondents preferred receiving health-related information through traditional media sources, such as television and radio, suggesting a need for accessible communication strategies, particularly for older demographics who may not engage with digital media extensively. Additionally, the influence of social circles, including family and neighbors, significantly shaped vaccine perceptions.

These findings highlight the importance of delivering consistent, evidence-based vaccine information through multiple communication channels that are tailored to different demographic groups. Although digital platforms have become increasingly important sources of health information, they have also facilitated the rapid spread of misinformation during the COVID-19 pandemic. Consequently, trusted healthcare professionals, public health institutions and community leaders remain critical sources of credible information that can strengthen public confidence and counter misinformation. Leveraging community-based communication, trusted HCWs and culturally appropriate messaging may therefore improve vaccine confidence and support future vaccination programs. This study demonstrated low levels of vaccine hesitancy and high COVID-19 vaccine acceptance among HCWs and nursing students, reinforcing their important role as advocates for vaccination within both healthcare settings and the wider community. Nevertheless, persistent knowledge gaps relating to vaccine safety, pregnancy, preventive measures following vaccination and perceived long-term effects indicate opportunities to strengthen vaccine education and professional development. Ensuring that HCWs have access to accurate, timely and evidence-based information regarding vaccine safety, effectiveness, eligibility criteria and evolving vaccination recommendations will further enhance their capacity to promote vaccine confidence and respond effectively to public concerns. Given that HCWS are among the most trusted sources of health information, strengthening their vaccine literacy is likely to have benefits extending beyond COVID-19 by supporting future immunization programs and preparedness for emerging infectious disease threats. While participants expressed varying degrees of concern regarding the COVID-19 pandemic, these findings primarily highlight the importance of maintaining public trust through transparent communication rather than indicating a need for specific mental health interventions. Continued investment in evidence-based risk communication, HCW education and public engagement will be essential for sustaining vaccine confidence during future public health emergencies.

**The public health implications of this study**

The high voluntary uptake of COVID-19 vaccination observed among HCWs demonstrates that strong vaccine acceptance can be achieved through evidence-based education, accessible vaccination services and trust in public health programs without necessarily relying on mandatory vaccination policies [22,24,25,27]. These findings highlight the important role of HCWs in protecting themselves, their colleagues and vulnerable patients while strengthening health system resilience during infectious disease outbreaks [22,24,66]. The willingness of HCWs to be vaccinated also has broader implications for other occupational vaccination programs, including seasonal influenza and hepatitis B vaccination, where HCWs serve as important advocates and role models for vaccine acceptance [15,19,22]. Public health programs should therefore continue promoting the benefits of vaccination while maintaining transparent, consistent and evidence-based communication to sustain confidence in routine and emergency immunization programs [12,42,51].

Despite the generally high vaccine acceptance observed in this study, persistent concerns regarding vaccine safety, long-term effects and vaccination during pregnancy indicate that vaccine confidence should not be regarded as static. Instead, HCW education should remain an ongoing process that adapts to evolving scientific evidence, emerging infectious disease threats and updated vaccination recommendations [28,43,52]. Although knowledge was not an independent predictor of voluntary vaccination in the multivariable analysis, descriptive findings identified important knowledge gaps relating to vaccine safety, pregnancy and preventive measures following vaccination. Educational interventions should therefore focus on improving vaccine literacy, strengthening confidence in the evidence base and equipping HCWs with the knowledge required to communicate effectively with patients and the public [22,24,25,27]. Continued reinforcement of complementary infection prevention measures alongside vaccination also remains important, particularly in the context of waning immunity, breakthrough infections and the emergence of new viral variants [28,50].

The findings also contribute to the ongoing debate surrounding mandatory vaccination within healthcare settings. Although voluntary vaccination was widely accepted by participants, differing views regarding mandatory vaccination highlight the need to balance occupational health responsibilities with respect for individual autonomy, informed consent and public trust [8,9,12]. The study does not provide evidence for recommending mandatory vaccination. Future occupational vaccination policies should prioritize voluntary, informed uptake and should consider compulsory measures only where a context-specific ethical and legal assessment demonstrates necessity, proportionality and the absence of comparably effective, less restrictive alternatives. Targeted communication strategies are particularly important for HCWS who remain uncertain about vaccination in special populations, including pregnant individuals and those with pre-existing medical conditions, where substantial evidence now supports vaccine safety and effectiveness [5,30].

Finally, ensuring equitable access to vaccination requires communication strategies that recognize demographic, educational and occupational differences within the healthcare workforce. Healthcare workers are frequently motivated by professional responsibility, altruism and the desire to protect vulnerable patients, colleagues and family members [66]. Strengthening vaccine literacy among HCWS is therefore likely to have benefits extending beyond individual protection by improving patient counseling, reinforcing public confidence in immunization programs and enhancing preparedness for future infectious disease emergencies. Collectively, these findings support the integration of evidence-based vaccine education, effective risk communication and occupational health policies as core components of resilient healthcare systems capable of responding to future public health threats [12,51].

**Limitations of the Study**

Although the study achieved a response rate of 73.8%, voluntary participation may have introduced non-response and self-selection bias. Individuals with stronger views, greater interest in vaccination, or greater access to institutional email may have been more likely to participate. The findings may therefore not fully represent the views of eligible students and staff who did not complete the questionnaire. This has the potential to restrict the applicability of the results to a wider community of HCWs. Most of the study participants are nursing students compared to academic and administrative staff. While nursing students undertake supervised clinical placements involving direct patient contact and share many occupational exposure risks with qualified HCWs, their differing professional status is an acknowledged limitation. The research depends on data that individuals describe about themselves, which might be influenced by memory bias or a tendency to provide socially desirable responses. Respondents may tend to exaggerate their favorable views towards vaccination or downplay their reluctance, which might result in potential errors in the findings. The study’s cross-sectional design hinders demonstrating causal correlations between variables. Although the research offers useful insights regarding vaccination attitudes and behaviors at a single moment, longitudinal studies would be necessary to evaluate changes over time and changes in attitudes and vaccination behavior over time.

The research was conducted at a single nursing institution, restricting the applicability of the results to other healthcare settings or areas. Healthcare infrastructure, cultural norms, and vaccination strategies in various situations may have varying effects on vaccination dynamics.

## 5. Conclusions

This study showed strong overall support for COVID-19 vaccination but highlighted ongoing concerns about safety and long-term effects. Strengthening vaccine literacy, maintaining transparent risk communication, and supporting voluntary, evidence-informed vaccination programs among healthcare workers will contribute to improved preparedness for future infectious disease emergencies.

Reporting voluntary rather than mandatory COVID-19 vaccination was not significantly associated with sex, marital status, vaccination status, previous COVID-19 infection, KAP, or reasons for vaccine hesitancy. However, occupation and age group were independently associated with the reported basis on which vaccination was received.

## Figures and Tables

**Table 1:** Biographical characteristics of the study respondents (*n* = 372).

Characteristics	Frequency (*n*)	Percentage (%)
**Respondents’ age**		
18–25 years	75	20.2
26–40 years	144	38.8
41–55 years	136	36.7
Above 55 years	16	4.3
Missing	1	0.3
**Sex of Health Workers**		
Male	70	18.8
Female	302	81.2
**Marital status**		
Single	252	67.9
Married	103	27.8
Divorced	6	1.6
Widowed	10	2.7
Missing	1	0.3
**Occupation**		
Nursing students	344	92.5
Academic staff	21	5.6
Admin and support staff	7	1.9
**Vaccination status (*n* = 370)**		
Fully vaccinated (including boosters)	104	28.1
Fully vaccinated (no boosters)	157	42.2
Vaccinated but awaiting 2nd dose	23	6.2
Not vaccinated	86	23.2
**Mandatory vs. voluntary vaccination (*n* = 275)**		
Mandatory	77	28.0
Voluntary	198	72.0
**COVID-19 infections (*n* = 371)**		
Yes (before vaccination/not vaccinated)	93	25.1
Yes (after vaccination)	52	14.0
No	226	60.9
**Primary media sources for information (*n* = 371)**		
Internet	88	23.9
Electronic media (TV, radio)	154	41.8
Social media	128	34.8
Print media (newspapers, local magazines, etc.	8	2.2
People (community, family members)	27	7.3

**Table 2:** Respondents’ knowledge regarding COVID-19 vaccination (*n* = 372).

No.	Knowledge Statements	Likert Scale Responses (*n*, %)			M (SD)
		Strongly Disagree/Disagree	Neutral	Strongly Agree/Agree	
K1	COVID-19 vaccines are effective at keeping you from getting COVID-19.	100 (27.2%)	109 (29.6%)	159 (43.2%)	3.15 (1.27)
K2	Employees maintain the regulations for preventing COVID-19 after vaccination (e.g., social distancing).	21 (5.8%)	20 (5.5%)	321 (88.7%)	4.34 (0.90)
K3	COVID-19 vaccine will also help keep you from getting seriously ill even if you get COVID-19.	90 (24.9%)	64 (17.7%)	208 (57.5%)	3.48 (1.29)
K4	During the COVID-19 pandemic, fully vaccinated individuals were subject to fewer movement and activity restrictions than those who were not fully vaccinated.	182 (50.3%)	53 (14.6%)	127 (35.1%)	2.74 (1.38)
K5	There is a website where you can apply for vaccination in South Africa.	26 (7.2%)	46 (12.7%)	290 (80.1%)	4.12 (0.95)
K6	Like all other vaccines, this vaccine has the potential for some side effects.	25 (6.7%)	20 (5.5%)	317 (87.6%)	4.32(1.19)
K7	Vaccines can be given to a person with pre-existing health conditions.	78 (21.5%)	60 (16.6%)	224 (61.9%)	3.57 (1.38)
K8	The vaccine can be given to pregnant women.	143 (39.4%)	67 (18.5%)	153 (42.3%)	2.99 (1.18)
K9	If there are side effects due to COVID-19 vaccination, they normally go away in a few days.	62 (17.1%)	61 (16.9%)	239 (66.0%)	3.69 (1.26)
K10	The COVID-19 vaccine can create long-term physical and health problems.	109 (30.1%)	102 (28.2%)	151 (41.7%)	3.21 (0.96)
	Knowledge construct				3.56 (1.78)

Key: M—mean, SD—standard deviation. Percentages are based on valid responses for each item and may not total 100% because of rounding and occasional missing responses.

**Table 3:** Respondents’ attitudes and perceptions towards COVID-19 vaccinations and reasons for hesitating to receive the vaccine.

No.	Attitude and Perception Statements	Likert Scale Responses (*n*, %)			M (SD)
		Strongly Disagree/Disagree	Neutral	Strongly Agree/Agree	
AP1	I feel personally concerned about the impact of the COVID-19 pandemic.	14 (3.9%)	34 (9.5%)	310 (86.6%)	4.26 (0.85)
AP2	I think that the COVID-19 vaccine is safe and effective.	51 (14.2%)	113 (31.6%)	194 (54.2%)	3.54 (1.06)
AP3	If I am eligible for the vaccine, I need to take it as soon as possible.	61 (17.0%)	85 (23.7%)	212 (59.2%)	3.59 (1.14)
AP4	I am aware that my family and neighbours should take the vaccine.	22 (5.9%)	48 (12.9%)	302 (81.2%)	4.11 (0.95)
AP5	I should motivate my family and neighbours to take the vaccine.	38 (10.2%)	64 (17.2%)	270 (72.6%)	3.92 (1.09)
AP6	I think vaccination will help us stop the spread of COVID-19.	48 (12.9%)	68 (18.3%)	256 (68.8%)	3.80 (1.10)
AP7	I think I’m not informed enough about vaccines.	182 (48.9%)	81 (21.8%)	109 (29.3%)	2.70 (1.27)
AP8	Are you hesitant to take the COVID-19 vaccine?	158 (42.5%)	89 (23.9%)	125 (33.6%)	2.89 (1.35)
AP9	Should the vaccine be mandatory for healthcare workers?	140 (40.3%)	50 (13.4%)	172 (46.2%)	3.08 (1.52)
AP10	Should the vaccine be mandatory for all South Africans?	165 (44.4%)	71 (19.1%)	136 (36.6%)	2.83 (1.45)
	Attitude and perception construct				3.47 (1.18)
	**Vaccine hesitancy statements**				
VH1	I don’t think I am eligible for it.	213 (57.3%)	75 (20.3%)	84 (22.6%)	2.48 (1.19)
VH2	I don’t know enough about it.	218 (58.6%)	66 (17,7%)	88 (23.7%)	2.51 (1.32)
VH3	I am afraid of the side effects.	160 (43.0%)	62 (16.7%)	150 (40.3%)	2.97 (1.34)
VH4	I may have to pay money for it.	305 (82.0%)	37 (9.9%)	30 (8.1%)	1.80 (1.02)
VH5	It is against my religious beliefs.	279 (75.0%)	50 (13.4%)	43 (11.6%)	2.00 (1.10)
VH6	Due to other reasons.	184 (50.3%)	60 (16.1%)	125 (22.6%)	2.70 (1.40)
	Hesitancy construct				2.41 (1.24)

Key: M—mean, SD—standard deviation.

**Table 4:** Multivariable logistic regression analysis of factors associated with reporting voluntary rather than mandatory COVID-19 vaccination.

Mandatory vs. Voluntary Vaccination	Adjusted Odds Ratio	95% Confidence Interval		*p*-Value
		Lower Bound	Upper Bound	
Sex (ref = male)	0.655	0.290	1.480	0.309
Marital status (ref = single/divorced)	0.847	0.551	1.301	0.448
Occupation (ref = nursing students)	2.289	1.083	4.838	0.030
Age group (ref = 18–40 years)	0.629	0.416	0.949	0.027
Vaccination status †	0.694	0.461	1.046	0.081
COVID-19 infection status †	1.010	0.722	1.415	0.953
Knowledge of COVID-19 vaccine (per one-unit increase)	0.962	0.667	1.389	0.837
Attitude and perceptions towards COVID-19 vaccine (per one-unit increase)	0.940	0.634	1.396	0.760
Reasons behind hesitating to take the COVID-19 vaccine (per one-unit increase)	0.878	0.632	1.218	0.435

Key: Ref—reference. † Vaccination status and previous COVID-19 infection were entered into the original model using their ordinal coding. Consequently, the odds ratios represent the change associated with a one-category increase in the coded variable and should be interpreted cautiously.

## Data Availability

The anonymized dataset supporting the findings of this study is available from the corresponding author upon reasonable request. IBM SPSS syntax used for statistical analyses is also available upon reasonable request.

## Supplementary Materials

The following supporting information can be downloaded at https://www.mdpi.com/article/10.3390/ijerph23081042/s1. Supplementary Materials File S1: Questionnaire.

## Abbreviations

The following abbreviations are used in this manuscript:

KAPs	Knowledge, Attitudes and Perceptions
HCWs	Healthcare Workers
COVID-19	Coronavirus Disease of 2019
HPV	Human Papillomavirus
EPI	Expanded Program on Immunization
OHSA	Occupational Health and Safety Act
SAHPRA	South African Health Products Regulatory Authority
KMO	Kaiser–Meyer–Olkin
