# Supplementary material for: A Cross-Sectional Study to Understand COVID-19 Vaccine Hesitancy Among Nursing Students and Health Workers: Implications for Future Outbreak Preparedness in South Africa
Source: Int J Environ Res Public Health. 2026 Aug 11;23(8):1042. doi: 10.3390/ijerph23081042 (PMC13513485; doi:10.3390/ijerph23081042)
Supplement: Supplementary file 1 [file ijerph-23-01042-s001.zip › ijerph-4427695-supplementary.pdf]

Questionnaire

Good Day, This survey will take 10 to 15 minutes. Your Contribution will be highly appreciated! In this survey, you will be asked some questions to evaluate your knowledge, attitude, and perception of the COVID-19 vaccination. Please read the questions carefully and provide your answers accordingly. This research has complied with the requisite ethical issues. Please note that all the information you provide is totally CONFIDENTIAL and will be used only by the research team for the purposes of the research. If you agree to participate in this survey, please continue to the next section. For further details, please contact the research team.

PLEASE ANSWER THE FOLLOWING QUESTIONS BY CROSSING (X) THE RELEVANT BLOCK

EXAMPLE: HOW TO COMPLETE THIS QUESTION.

Your marital status?

If you're single:

Married	
Single	X
Divorced	
Widow	

1. Are you above 18 years of age?

Yes	
No	

2. Are you employed or registered as student at Mpumalanga College of Nursing?

Yes	
No	

3. Have you read the Information sheet provided for the study and do you understand it and provided informed consent for data to be collected from you?

Yes	
No	

NB: If you choose NO from the above questions you cannot continue with the survey

<u>Section A: Socio-demographic</u>

This section of the questionnaire refer to background or demographic information. The information will allow me to compare groups of respondents. As we are aware of the sensitivity of the questions in this

section, please note that all the information you provide is totally CONFIDENTIAL and will be used only by the research team for the purposes of the research. Your co-operation is appreciated.

1. What is your Gender?

Male	
Female	
Non-binary	
Prefer not to say	

2. What is your Marital Status?

Single	
Married	
Divorced	
Widow	

3. What is your position at Mpumalanga College of Nursing?

Student	
Admin & Support Staff	
Academic Staff	

4. What is your age range?

18 to 25 years	
26 to 40 Years	
41 to 55 Years	
Above 55 Years	

5. Are you Vaccinated against COVID-19?

Yes, Fully Vaccinated against COVID-19, Including Boosters	
Yes, Fully Vaccinated against COVID-19, No Boosters	
Yes, Vaccinated against COVID-19, But Awaiting Second Dose	
No, Not vaccinated at all against COVID-19	

Please Indicate Date of First dose ______________________

6. If you choose YES on above question was it mandatory or voluntary?

Mandatory	
Voluntary	

7. Have you ever being infected with COVID-19?

Yes, (before my vaccination/not vaccinated)	
Yes, (even after I was vaccinated)	
No	

8. Which media you use most for COVID-19 vaccine and precautionary measures related information?

Internet	
Electronic media (TV, Radio)	
Social Media	
Print media(newspaper, local Magazines etc	
People (community, family members)	

Section B: Assessment of Knowledge

This section explores your Knowledge towards COVID-19 Vaccine and to what extent you agree with each of the following statements. Please indicate your answer using five (5) Likert scale options: Strongly Agree [SA], Agree [A], Neutral [N], Disagree [D] and Strongly Disagree [SD].

	SA	A	N	D	SD
9. COVID-19 vaccines are effective at keeping you from getting COVID-19.					
10. You need to maintain the regulations of preventing COVID-19 after being vaccinated (e.g social distancing).					
11. Getting a COVID-19 vaccine will also help keep you from getting seriously ill even if you get COVID-19.					
12. During the COVID-19 pandemic, fully vaccinated individuals were subject to fewer movement and activity restrictions than those who were not fully vaccinated.					
13. There is a website where you can apply for COVID-19 vaccination in South Africa.					
14. Like all other COVID-19 vaccines, this vaccine has the potential for some side effects.					

15. COVID-19 Vaccine can be given to a person with pre-existing health conditions					
16. The COVID-19 vaccine can be given to pregnant women.					
17. If there are side effects due to COVID-19 vaccination, they normally go away in a few days.					
18. The COVID-19 vaccine can create long term physical and health problems.					

Section C: Assessment of Attitude and Perception

This section explores your Attitude and Perception towards COVID-19 Vaccine and to what extent you agree with each of the following statements. Please indicate your answer using five (5) Likert scale options: Strongly Agree [SA], Agree [A], Neutral [N], Disagree [D] and Strongly Disagree [SD].

	SA	A	N	D	SD
19. I feel personally concerned about the impact of the COVID-19 pandemic.					
20. I think that the COVID-19 vaccine is safe and effective.					
21. If I am eligible for the vaccine, I need to take it as soon as possible.					
22. I am aware that my family and neighbours should take the vaccine.					
23. I should motivate my family and neighbours to take the vaccine.					
24. I think vaccination will help us to stop the spreading of COVID-19.					
25. I think I'm not informed enough about vaccines.					
26. Are you hesitant to take the COVID-19 vaccine?					
27. Should the vaccine be mandatory for healthcare workers?					
28. Should the vaccine be mandatory for all South Africans?					

Section D: Reason behind hesitation to take COVID-19 Vaccine

This section explores your Hesitation towards COVID-19 Vaccine and to what extent you agree with each of the following statements. Please indicate your answer using five (5) Likert scale options: Strongly Agree [SA], Agree [A], Neutral [N], Disagree [D] and Strongly Disagree [SD].

	SA	A	N	D	SD
29. I am hesitant to take the COVID-19 vaccine because I don't think I am eligible for it.					
30. I am hesitant to take the COVID-19 vaccine because I don't know enough about it.					

31. I am hesitant to take the COVID-19 vaccine because I am afraid of the side effects.					
32. I am hesitant to take the COVID-19 vaccine because I may have to pay money for it.					
33. I am hesitant to take the COVID-19 vaccine because it is against my religious belief.					
34. I am hesitant to take the COVID-19 vaccine because of other reasons.					

Thank you for participating in the study.
